# Methods to investigate intrathecal adaptive immunity in neurodegeneration

**DOI:** 10.1186/s13024-021-00423-w

**Published:** 2021-01-22

**Authors:** Hamilton Oh, Olivia Leventhal, Divya Channappa, Victor W. Henderson, Tony Wyss-Coray, Benoit Lehallier, David Gate

**Affiliations:** 1 Department of Neurology and Neurological Sciences, Stanford University School of Medicine, Stanford, California USA; 2 Epidemiology and Population Health, Stanford University School of Medicine, Stanford, California USA

**Keywords:** Cerebrospinal fluid cells, CSF, Intrathecal cells, Neurodegeneration, Adaptive immunity, T cells, T cell receptor (TCR), Antigen

## Abstract

**Background:**

Cerebrospinal fluid (CSF) provides basic mechanical and immunological protection to the brain. Historically, analysis of CSF has focused on protein changes, yet recent studies have shed light on cellular alterations. Evidence now exists for involvement of intrathecal T cells in the pathobiology of neurodegenerative diseases. However, a standardized method for long-term preservation of CSF immune cells is lacking. Further, the functional role of CSF T cells and their cognate antigens in neurodegenerative diseases are largely unknown.

**Results:**

We present a method for long-term cryopreservation of CSF immune cells for downstream single cell RNA and T cell receptor sequencing (scRNA-TCRseq) analysis. We observe preservation of CSF immune cells, consisting primarily of memory CD4^+^ and CD8^+^ T cells. We then utilize unbiased bioinformatics approaches to quantify and visualize TCR sequence similarity within and between disease groups. By this method, we identify clusters of disease-associated, antigen-specific TCRs from clonally expanded CSF T cells of patients with neurodegenerative diseases.

**Conclusions:**

Here, we provide a standardized approach for long-term storage of CSF immune cells. Additionally, we present unbiased bioinformatic approaches that will facilitate the discovery of target antigens of clonally expanded T cells in neurodegenerative diseases. These novel methods will help improve our understanding of adaptive immunity in the central nervous system.

**Supplementary Information:**

The online version contains supplementary material available at 10.1186/s13024-021-00423-w.

## Background

The cerebrospinal fluid (CSF) provides insight into brain physiology of living individuals. Biochemical analysis of CSF is routinely utilized as a diagnostic tool in neurodegeneration [1–3]. For example, in Alzheimer’s disease (AD), changes in protein levels of tau and amyloid-β are indicative of disease pathology. However, while CSF protein biomarkers guide diagnosis of patients with neurodegenerative diseases, the cells patrolling the interstitial fluid are often centrifuged and discarded. Only in cases of extreme central nervous system inflammation, such as meningitis or encephalitis, are CSF cells utilized as a diagnostic. Blood cells, on the other hand, are routinely used to assess health and disease. We surveyed the literature to gain insight into CSF cell composition and summarized the main studies (Table 1). The CSF is primarily composed of antigen-experienced memory CD4^+^ and CD8^+^ T cells, plasmacytoid dendritic cells (DCs), and CD56^high^ natural killer (NK) cells. Naïve T cells, monocytes, granulocytes, myeloid DCs, basophils, and B cells are not as abundant in the CSF as they are in peripheral blood [2]. Moreover, the cellular density of CSF (1000–3000 cells per mL) is drastically diluted compared to blood (millions of cells per mL) [1, 4, 5].

**Table 1:**
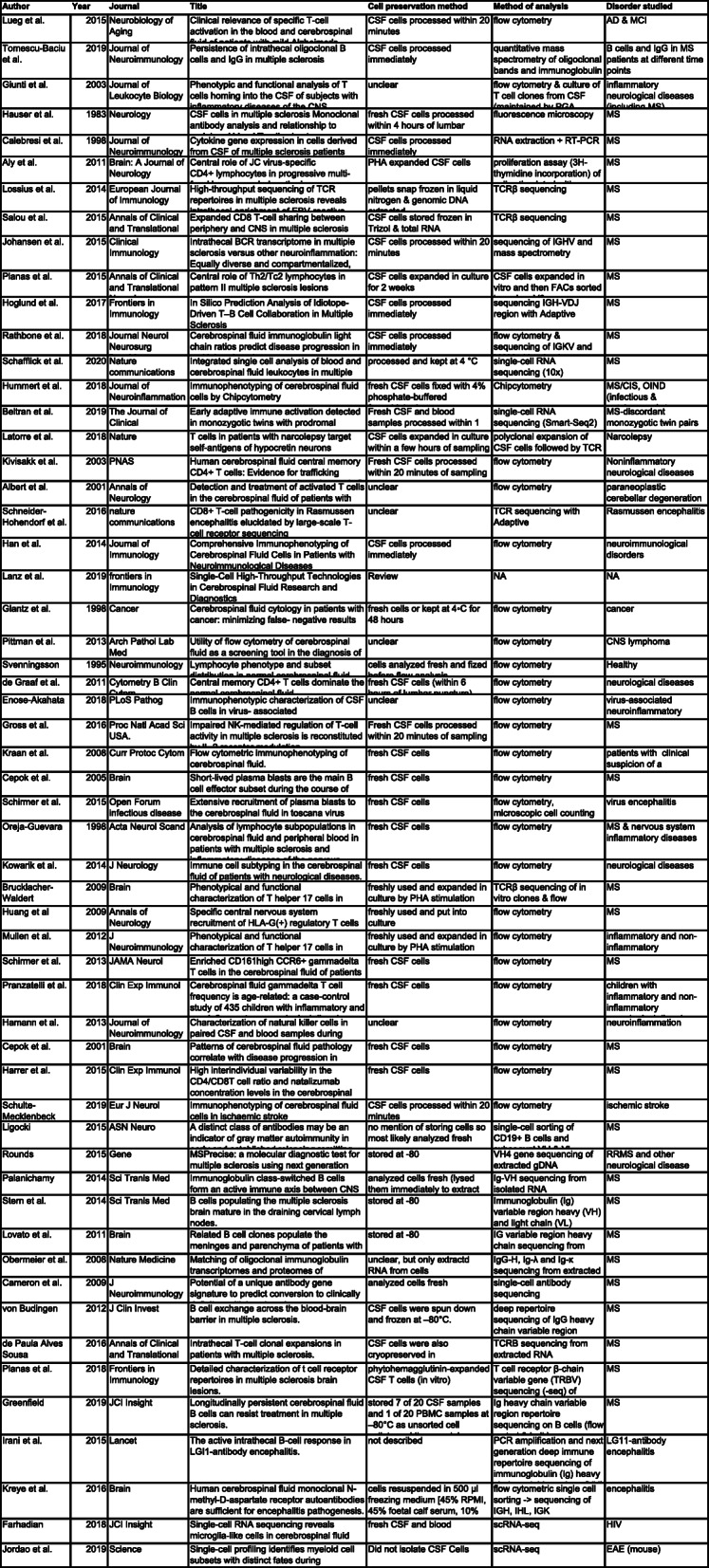
Studies focused on CSF cells

An exhaustive search was performed on pubmed.gov to include all studies focused on CSF cell composition in disease

To our knowledge, only the multiple sclerosis field has rigorously evaluated live CSF cells to assess disease [1, 5–14]. However, recent studies provide evidence of CSF T cells in the pathobiology of other neurodegenerative diseases. These include the discovery of hypocretin-specific CD8^+^ T cells in narcolepsy [15] and T cell clones shared between Rasmussen’s encephalitis patients [16]. Moreover, a recent report identified α-synuclein-specific T cells in peripheral blood of Parkinson’s disease (PD) patients [17], but whether these cells enter the CSF is unknown. Finally, our group reported the presence of clonally expanded T cells in the CSF of AD patients and granzyme-A^+^ CD8^+^ T cells in the parenchyma of AD brains [18]. Cumulatively, these advances provide evidence that T cells may be involved in the pathobiology of several neurodegenerative diseases and emphasize the value of CSF cells to study the role of adaptive immunity in neurodegenerative disease.

Most of the aforementioned studies have relied on freshly isolated CSF cells [1, 5, 6, 9–20] or extraction of genomic DNA or RNA from frozen cells [7, 8]. Analyzing fresh CSF cells provides the highest number of viable cells and the closest approximation of their endogenous physiology, but introduces batch effects which are especially important to avoid in high-throughput sequencing experiments. Conversely, cryopreservation allows for parallel analysis of multiple samples acquired longitudinally, minimizing batch effects and reducing total time spent. Increased evidence of CSF cell involvement in neurodegenerative disease warrants a standardized approach for the long-term preservation of CSF immune cells and also calls for methods to assess the role of CSF immune cells in pathobiology. Here, we report a method for the long-term storage and subsequent analysis of CSF cells by single cell RNA and T cell receptor sequencing (scRNA-TCRseq). Moreover, we present unbiased bioinformatics approaches to identify and visualize disease-associated TCRs. These methods will enable researchers to discover novel disease mechanisms in neurodegenerative diseases, particularly in the context of adaptive immunity.

## Results

**Fig. 1:**
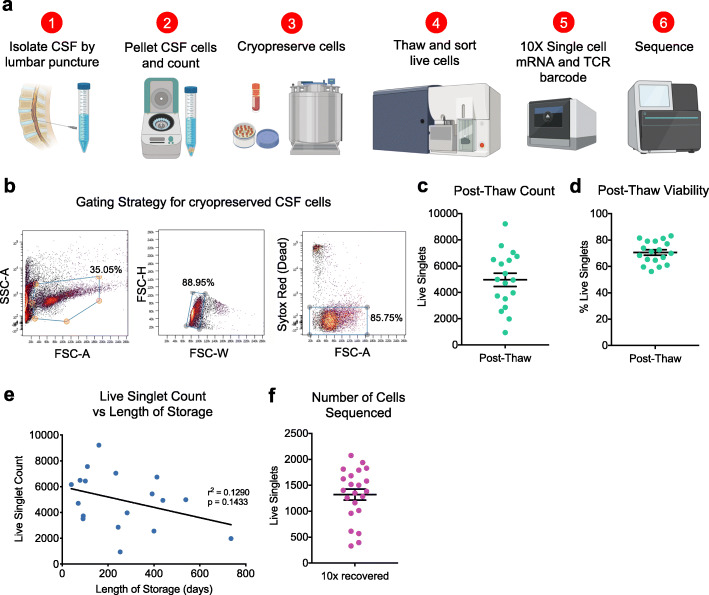
Cryopreservation of human cerebrospinal fluid cells retains cellular viability. **a** Workflow for cryopreservation of CSF cells for scRNA-TCRseq. **b** Gating strategy for sorting live cryopreserved cells by flow cytometry. **c** Quantification of live cell count (live singlets) from flow cytometry sorting following cell thawing. Mean ± s.e.m. **d** Quantification of viability (percentage of live singlets out of all singlets) from sorting by flow cytometry. Mean ± s.e.m. **e** Linear regression of live cell count post thaw versus length of storage. **f** Quantification of the number of CSF cells captured for sequencing

We developed a standardized workflow for the isolation and cryopreservation of CSF immune cells for scRNA-TCRseq (Fig. 1a). Following extraction of CSF by lumbar puncture, cells were pelleted by centrifugation, checked for blood contamination (Supplementary Fig. 1a), and cryopreserved. Samples without blood contamination were thawed at 37 °C and live cells were sorted by flow cytometry (Fig. 1b), resulting in an average of 4961 live cells per sample (Fig. 1c) and 71% viability of singlets (Fig. 1d). Length of storage had no significant impact on number of cells acquired after thaw (Fig. 1e).

**Fig. 2:**
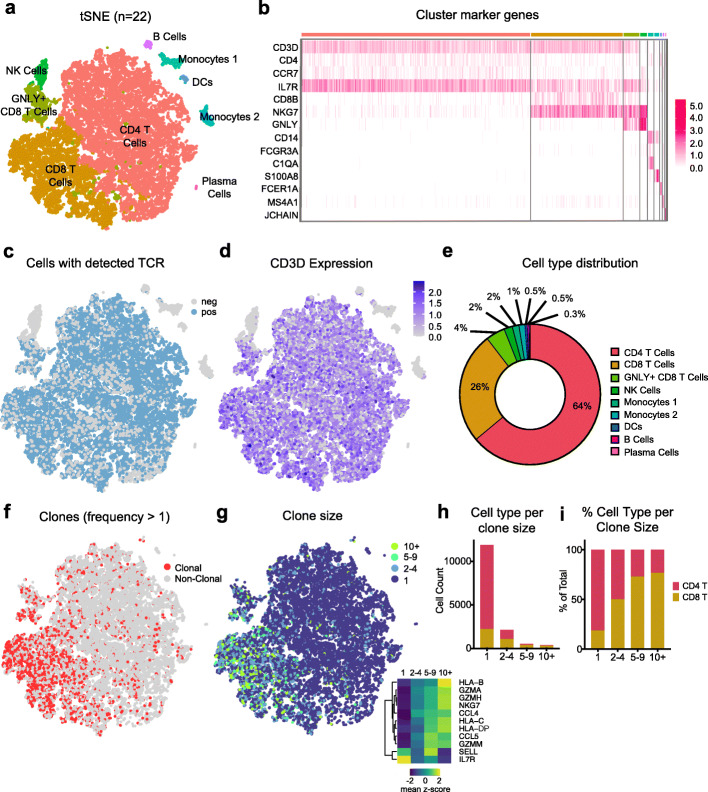
scRNA-TCRseq of human cerebrospinal fluid reveals clonally expanded CD8^+^ T cells. **a** Seurat dimensionality reduction and clustering of 22 CSF samples that passed quality control displayed on tSNE (GNLY: Granulysin; DCs: Dendritic Cells). **b** Heatmap of cluster marker genes used to annotate clusters. **c** Cells with detected TCR displayed on tSNE. **d** CD3D expression displayed on tSNE. **e** Quantification of average cell type distribution based on Seurat clustering. **f** Clones – cells with TCR sequences shared with other cells – displayed on tSNE. Only cells with detected TCRs are included. **g** Clones of different sizes displayed on tSNE. Only cells with detected TCRs are included; lower right heatmap shows significant differentially expressed genes between clone size bins. **h** Quantification of number of T cell types per clone size. Only cells with detected TCR are included. GNLY^+^CD8^+^ T Cells and CD8^+^ T cells were combined as CD8^+^ T cells. **i** Quantification of % T cell types per clone size

Here, we sequenced CSF cells from a total of 24 subjects – 8 healthy controls, 4 patients with clinical AD, 5 with mild cognitive impairment (MCI), and 7 with PD (Table 2). After thawing and sorting live cells, we prepared single cell libraries then amplified the global transcriptome and TCRαβ genes from each sample. This resulted in an average of 1323 sequenced cells per sample (Fig. 1f). After quality control (Supplementary Fig. 2a) and batch correction, dimensionality reduction and clustering revealed separation of CD8^+^ and CD4^+^ T cells, NK cells, monocytes, DCs, B cells, and plasma cells (Fig. 2a), based on marker gene expression analysis (Fig. 2b). Cells with sequenced TCRs (Fig. 2c) overlapped with cells that expressed the pan-T cell marker CD3D (Fig. 2d), confirming T cell identity and productive TCR sequencing. Importantly, we did not detect platelet genes, such as *PPBP*, among our CSF cells, confirming that our samples were not contaminated with blood. Additionally, clusters were not enriched for particular subjects, diagnosis, processing batch or sequencing batch (Supplementary Fig. 2b-e). After clustering and annotating cells, we calculated the average proportion of each cell type in our CSF samples (Fig. 2e). CD4^+^ T cells were the most abundant cell type (64%) followed by CD8^+^ T Cells (30%), myeloid cells (3.5%), NK cells (2%), plasma cells (0.5%) and B cells (0.3%) (Fig. 2e). There was no significant difference in the cell type composition between disease groups (Supplementary Fig. 2f). These results indicate that our method of long-term preservation of CSF cells retains several immune cell populations and is suitable for downstream scRNA-TCRseq analysis.

**Table 2:**
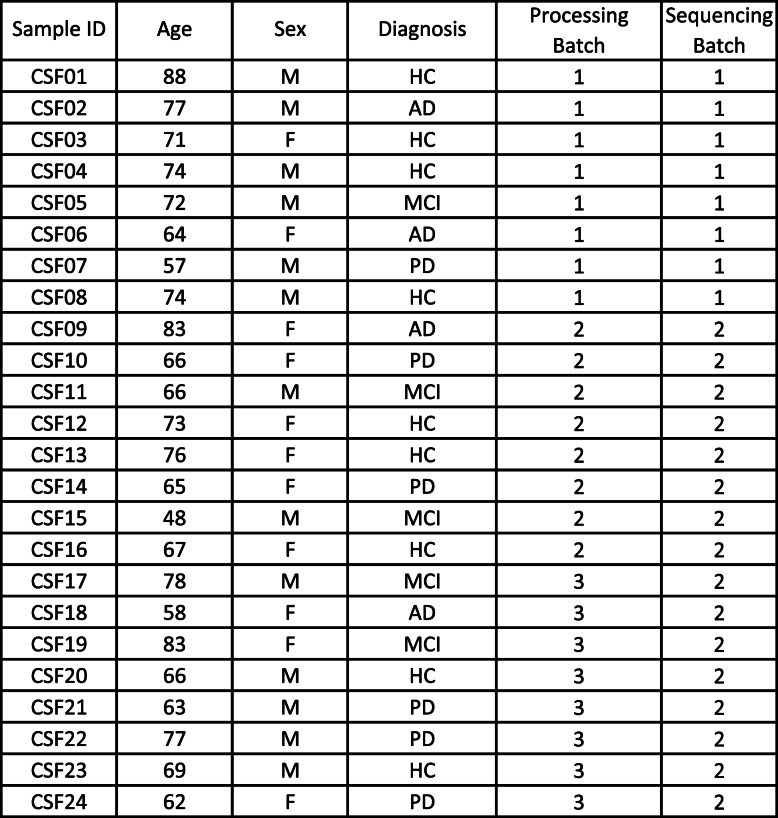
Metadata for samples used in scRNA-TCRseq

Subject characteristics for CSF samples utilized in this study

We next sought to develop methods to facilitate the discovery of TCR-antigen specificities relevant to neurodegenerative diseases. We first assessed whether clonally expanded T cells were present in the CSF, as determined by identification of cells with identical TCR sequences. Indeed, we identified numerous clonally expanded T cells (Fig. 2f) and observed that larger clones (of size > 5) were enriched in CD8^+^ T cells compared to CD4^+^ T cells (Fig. 2g-i). These larger CD8^+^ T cell clones showed increased expression of genes related to cytotoxicity (*GZMA*, *GZMH*, *GZMM*, *NKG7*), cytokine signaling (*CCL4*, *CCL5*) and antigen presentation (*HLA-B*, *HLA-C*, *HLA-DP*) as well as decreased expression of genes associated with naïve T cells (*SELL*) and CD4^+^ T cells (*IL7R*) (Fig. 2g). Interestingly, the expression levels of these genes appeared to follow a gradient based on clone size, showing that our method of CSF preservation retains subtle gene expression patterns within cell types.

**Fig. 3:**
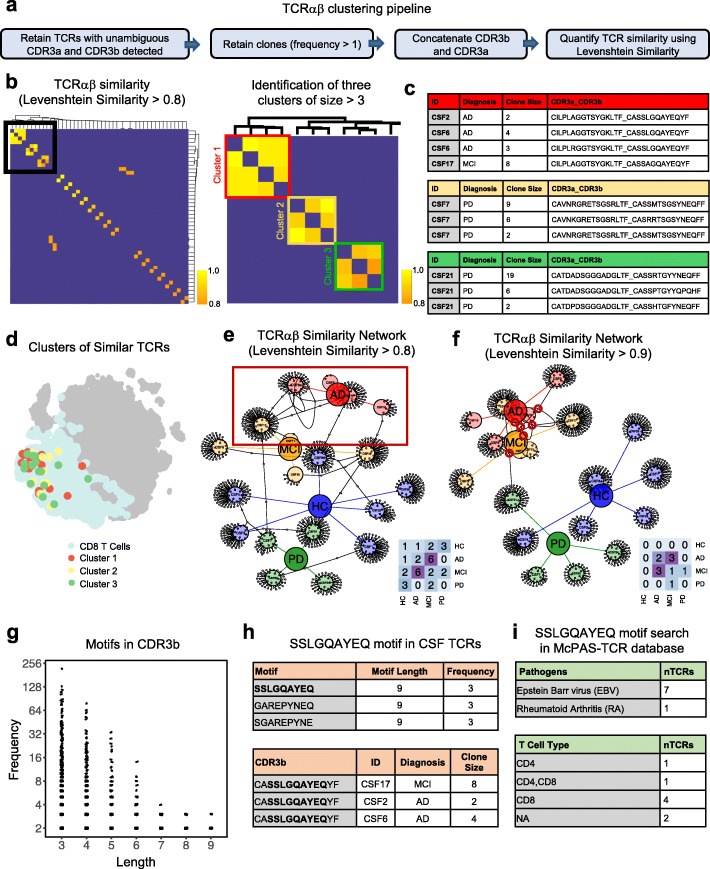
Analysis of T cell receptor sequence similarity within and between neurodegenerative disease groups. **a** Workflow for quantifying TCRαβ similarity. Clonal TCRs with unambiguous CDR3a and CDR3b sequences were retained for analysis. L-sim values were then computed between all possible combinations of TCRαβ sequences. **b** At left: heatmap highlighting TCR combinations with L-sim score > 0.8. At right: inset of heatmap shows three clusters of similar TCRs. Color bar represents L-sim score. **c** Metadata of clustered TCRs shown in b). Note the similarity of TCRs within disease groups. **d** Three clusters of similar TCRs identified in c) highlighted on tSNE. Clusters of TCRs overlap with CD8^+^ T cells. **e** TCR network displaying connections between samples with similar sequences (L-sim > 0.8) identified in b). Each node is a unique patient sample with each small circle sprouting off a node representing a unique clonal TCR. Nodes are colored by disease group; lower right heatmap shows number of similar TCRs between unique samples per disease group. **f** TCR network (L-sim > 0.9). **g** Quantification of shared motifs present in TCRβ sequences. Clonal TCRs with unambiguous CDR3b sequences were retained for analysis. The first and last two amino acids were removed from the analysis, since these regions are highly conserved. **h** Table of motifs of length 9. The SSLGQAYEQ motif is highlighted and metadata corresponding to patient samples is shown. **i** Upper: table of pathogens specific to SSLGQAYEQ motif based on search of the public McPAS-TCR database. Lower: table of SSLGQAYEQ motif-containing T cell types based on public McPAS-TCR database search

Given the abundance of clonally expanded T cells in the CSF, we reasoned that examining TCR similarity within and between disease groups could provide insight into disease-relevant TCR-antigen specificities. We first used a Levenshtein similarity (L-sim) score to quantify TCR similarity. This score is based on the Levenshtein distance algorithm, which calculates the number of edits, deletions, or insertions required to make two strings identical. L-sim includes a string length normalization and transformation of the final value to be between 0 and 1, with 1 representing identical TCR sequences. To examine TCR similarity within and between disease groups, we calculated the L-sim score for each possible pair of TCRs in our dataset, including healthy controls. To increase the confidence of this analysis and to focus on disease-relevant TCRs, we filtered our dataset to only include complete, unambiguous TCRαβ sequences from clonally expanded T cells (Fig. 3a). Intriguingly, L-sim scores followed a normal distribution with very few highly similar TCRs in the dataset (Supplementary Fig. 3a). By selecting TCRs with L-sim > 0.8, we identified several clusters of similar TCRαβ sequences (Fig. 3b). Interestingly, the largest clusters contained TCR sequences exclusively from patients with neurodegenerative diseases, even though healthy control TCRs were also included in the analysis (Fig. 3c). Notably, disease-associated clonal TCRs were expressed by CD8^+^ T cells (Fig. 3d). Moreover, three of the TCRβ sequences in Cluster 1 were identical (CASSLGQAYEQYF; Supplementary Fig. 3b) and have been shown to be specific for Epstein-Barr virus (EBV) epitope EBNA3A [18].

To more broadly understand TCR similarity within and between disease groups, we developed a TCR node network visualization that displays connections between similar TCRs (L-sim > 0.8) organized by unique samples and by disease groups. This analysis revealed that AD and MCI patients have numerous similar TCR sequences, while PD and healthy control subjects share fewer similar TCR sequences (Fig. 3e). Visualizing TCR similarity using a stricter L-sim cutoff of 0.9 showed that highly similar TCRs in our dataset were almost exclusively shared within AD/MCI patients, and no similar TCRs were shared with healthy controls (Fig. 3f). This suggests that AD and MCI patients may have similar T cell antigen specificities in the CSF, perhaps indicative of a disease-specific adaptive immune response. To validate our node network method, we utilized a ground-truth dataset to identify candidate TCRs involved in a separate neurodegenerative disease. We generated TCR networks from publicly available CSF TCRβ sequences from narcolepsy patients. This dataset contains experimentally determined TCRβ sequences that target hypocretin (HCRT) and Tribbles Pseudokinase 2 (TRIB2) antigens [15]. We identified several identical TCRβ sequences shared between unique narcolepsy CSF CD4^+^ T cell samples. Interestingly, we also found a pair of identical TCRβ sequences (one being derived from CSF and another from blood) that was previously determined to be HCRT-specific [15] (Supplementary Fig. 4a). Moreover, we identified a candidate HCRT-specific TCRβ sequence shared between three unique narcolepsy CSF CD8^+^ T cell clones (Supplementary Fig. 4b). Interestingly, TCRs from narcolepsy did not show overlap with TCRs from healthy controls, suggesting that these TCRs are relevant and specific to narcolepsy. Cumulatively, using an independent TCR dataset, we validate L-sim and TCR network visualization as tools to uncover disease-relevant TCRs.

In addition to L-sim, we show that motif analysis can be used to identify TCR sequences that share antigen specificity within and between disease groups. Using a sliding window approach, we quantified the frequency of every motif of every size from our pool of TCRβ sequences (Fig. 3g). We then inspected antigen specificity of large, frequent motifs by searching the motifs in the McPAS-TCR specificity public database [21]. This search uncovered motifs which were highly correlated with specific antigens, including the SSLGQAYEQ motif, which was found only in AD or MCI patients (Fig. 3h). This motif is expressed primarily by CD8^+^ T cells with specificity against EBV (Fig. 3i). In summary, we show that TCR motif analysis can be used to identify disease-associated TCRs, as well as their established antigen targets.

## Discussion

Most studies performed on human cells rely on peripheral blood mononuclear cells. However, utilizing peripheral cells as a read-out of immune changes in neurodegeneration limits our ability to understand central immunity. CSF cells, on the other hand, provide a way to directly study immunology in the central nervous system. Indeed, recent studies of intrathecal immunity by us and others have shown this understudied immune compartment to be relevant to the pathobiology of neurodegenerative diseases [1, 5–20, 22]. Yet, CSF cells have been widely understudied because of the invasive method of CSF extraction (typically via lumbar puncture), and because cells are often discarded for proteomic analysis. Cells not discarded are typically analyzed fresh, given the delicate nature of CSF cells [23]. However, independent processing of samples can lead to significant batch effects, which are of particular concern in high-throughput sequencing experiments. Therefore, to allow for multiple samples to be processed simultaneously, we developed a method for long-term preservation of CSF cells.

Our simple and quick method allows for the preservation of an average 4961 live cells after thawing, with no significant effect of storage length on the number of cells acquired post thaw. We find 64% of intrathecal cells are CD4^+^ T cells and 30% are CD8^+^ T cells, compared to 60–83% and 11–20%, respectively, as reported by others [1, 20]. We find that monocytes, DCs, and NK cells combined make up 6% of CSF cells, whereas other studies report that monocytes make up 5–12%, DCs less than 4%, and NK cells around 5% of fresh CSF cells [1]. We also detected small proportions of plasma cells (0.3%) and B cells (0.5%), which aligns with the less than 1% of B cells previously reported in fresh CSF [22]. The differences in CSF composition between this study and previous studies may be explained by the increased susceptibility to cell loss of myeloid cells compared to lymphocytes from freshly isolated CSF samples, [23] combined with the added stress of the freeze and thaw process. Overall, while we find a slight reduction in the number of recovered myeloid cells and NK cells, our method preserves T cells, the most abundant cell type in fresh CSF. Preservation of these cells enables the molecular study of adaptive immunity in neurodegenerative disease by scRNA-TCRseq while minimizing batch effects and time restrictions.

Beyond introducing a method to store human CSF cells, we show that scRNA-TCRseq analysis can provide insight into the TCR repertoire and antigen specificities of clonally expanded T cells within and between neurodegenerative disease groups. We provide an easy-to-use and readily modifiable R script that can act as a base template for future studies. By quantifying the similarity of each TCR to every other TCR using the L-sim algorithm and displaying similar TCRs in a node network, we show that TCRs from patients with neurodegenerative diseases, namely AD and MCI, cluster together. We also validate L-sim and TCR network visualization using an experimentally determined HCRT-specific TCRβ sequence in narcolepsy CSF. This approach also identified several candidate HCRT-specific CD4^+^ and CD8^+^ T cell TCRβ sequences. Additionally, we show that motif analysis can be used to identify TCRs that may share antigen specificity. Both similarity analyses independently revealed clusters of TCRs in patients with neurodegenerative diseases that were specific to EBV. However, these clusters represent only a small fraction of the TCR repertoire in the CSF. Moreover, our current knowledge of TCR antigen specificity is largely limited to studies of infectious disease, thus biasing results to viral epitopes.

It will be important to continue to expand our knowledge of TCR antigen specificity in neurodegenerative diseases, especially in diseases where infection is not thought to be the primary cause. This is especially important given the growing evidence that T cells may negatively contribute to the pathobiology of several neurodegenerative diseases, including AD and PD [17, 18]. Utilizing these methods to identify candidate disease-associated TCRs will aid in the identification of self/non-self antigen targets of clonally expanded T cells in neurodegenerative diseases.

## Conclusions

In conclusion, we provide a detailed method for long-term preservation of CSF cells and subsequent analysis by scRNA-TCRseq to study clonally expanded T cells in neurodegenerative disease. Importantly, we uncover several disease-associated TCR clusters of unknown specificity. Although these TCRs are highly similar, additional studies will need to confirm their shared specificity for antigens. Future studies utilizing high throughput peptide screens, such as yeast surface display platforms, could be employed to identify novel antigens. Theoretically, these novel antigens could serve as novel therapeutic targets or biomarkers for neurodegenerative diseases. With increasing innovation and throughput in technologies that aid in the identification of TCR-antigen specificities, the mapping of clonally expanded TCRs will improve our understanding of the role of T cells in the pathobiology of neurodegenerative diseases. Ultimately, we anticipate that these methods will aid in the development of novel adaptive immune-focused treatments for neurodegenerative disease.

## Methods

### Study participants

Samples were acquired through the Stanford Brain Rejuvenation Program, the NIA funded Stanford Alzheimer’s Disease Research Center (ADRC), the University of California at San Francisco ADRC and the University of California at San Diego ADRC. Collection of CSF was approved by the Institutional Review Board of each university; written consent was obtained from all subjects. A total of 34 living subjects were used in this study, 24 of which were used for scRNA-TCRseq. The 24 subjects included 8 healthy controls, 4 patients with AD, 5 with MCI, and 7 with PD.

### Cryopreservation of CSF cells

CSF was collected by lumbar puncture, then centrifuged at 300 *rcf* for 10 min at 4 °C to pellet immune cells. Importantly, CSF samples were checked for blood contamination by examining the pellet for the presence of red blood cells by eye. An example of a CSF sample contaminated with blood is shown in Supplemental Fig. 1a. Note that cells should remain at 4 °C until they are further processed, but it is best to freeze the cells as quickly as possible to limit cell death. The supernatant (cell-free CSF) was aliquoted, carefully leaving behind 100 μl of CSF with the pelleted cells. One hundred μl of CSF was left so that cells were concentrated enough for counting and viability measurements. The pelleted cells were then gently resuspended in the 100 μl CSF and 10 μl of resuspended cells were then removed for counting. Importantly, cells were gently resuspended by first tapping the bottom of the tube and then gently triturating 10 times, making sure not to touch the pipette tip to the edge of the tube. Then, 10 μl of CSF was removed and mixed with 10 μl trypan blue to assess red blood cell content and viability. Cells were then visualized on a TC20 automated cell counter (BioRad) and cell number, viability and the presence or absence of red blood cells was recorded. CSF samples contaminated with blood were discarded. The resuspended cells were then mixed with 900 μl Recovery Cell Culture Freezing Medium (Thermo Fisher Scientific). This medium is an optimized version of the typical freezing medium, containing high-glucose Dulbecco’s Modified Eagle Medium with 10% serum and 10% dimethyl sulfoxide. We utilized this medium because it is quality tested for pH, osmolality, sterility, and endotoxin and each lot is quality tested on CHO-K1 cells. The freezing medium was first thawed at 37 °C, aliquoted, and stored at − 20 °C. Before use, the medium was thawed at 37 °C and kept on ice. After each aliquot was thawed, the freezing medium was stored at 4 °C for up to one month. All samples were frozen overnight at − 80 °C in a Mr. Frosty freezing container (Thermo Fisher Scientific) and transferred the following day to liquid nitrogen for long-term storage. CSF cells were stored in liquid nitrogen for an average of 266 days.

### Preparation of frozen CSF cells for analysis

CSF cells were thawed at 37 °C in a water bath with the media submerged and the top of the tube out of the water. Cells were kept in the water bath for as little as possible and removed when the media was nearly completely thawed. The cells were then removed and gently pipetted into a 5 mL flow cytometry tube containing 3 mL pre-warmed (37 °C) sorting buffer (PBS with 0.04% bovine serum albumin). The tube was then rinsed once with the sorting buffer and placed into the same flow cytometry tube. Cells were then centrifuged at 350 *rcf* for 10 min. The supernatant was removed, and cells were resuspended in 500 μl sorting buffer. ½ μl of Sytox Red (Thermo Fisher Scientific) was added to the sample immediately before sorting by flow cytometry. Live cells were sorted into 1.5 mL Eppendorf tubes containing 750 uL sorting buffer. Once all samples were sorted, cells were spun at 350 *rcf* at 4 °C in a spinning bucket centrifuge for 7 min. The supernatant was then removed, leaving behind 10 μl. Ten μl was left behind to resuspend the CSF cells and load the entire volume for droplet-based scRNA-seq.

### Drop-seq of CSF cells

Chromium Single Cell 5′ Library & Gel Bead kit, Chromium Single Cell 5′ Library construction kit, Chromium Single Cell A Chip Kit, and Chromium i7 Multiplex kit (10X Genomics) were used for scRNAseq of CSF cells. We followed 10x Genomic’s User Guide for library construction. The only change we made to their protocol was in Step 1, GEM Generation & Barcoding. The user guide recommends loading a certain volume of cell suspension stock depending on the concentration of the cell suspension and the user’s desired cell recovery. However, because CSF contains such low cell numbers, we loaded all the cells that were resuspended in 10 μl of sorting buffer. We then added the 10 μl of cell suspension and 21.7 μl of nuclease free water, which results in the same total volume of cell suspension/water that the user guide recommends. After library construction, libraries were sequenced by Novogene on a Novoseq S4 sequencer and FASTQ files were generated by Novogene. Cell Ranger v.3.0.2 was used to generate gene-expression matrices for CSF cells. Reads from the 10X v.2 5′ paired library were mapped to the human genome build GRCh38 3.0.0. The 5′ gene-expression libraries were then analyzed with the Cell Ranger count pipeline and the resulting expression matrix was used for further analysis in the Seurat package v.3.0.

### Seurat clustering of CSF cells

Individual sample expression matrices were loaded into R using the function Read10x under the Matrix package v.1.2–15. The expression matrix for each sample was merged into one Seurat object using the CreateSeuratObject and MergeSeurat functions. The Seurat package v.3.0 was used for filtering, variable gene selection, normalization, scaling, dimensionality reduction, clustering and visualization. Genes were excluded if they were expressed in fewer than 10 cells and cells were excluded if they expressed fewer than 200 genes. Cells that expressed more than 1600 genes, more than 6000 unique molecular identifiers (UMIs) and more than 10% mitochondrial genes (indicative of cell death) were excluded from the analysis. The sctransform normalization method was used to normalize, scale, select variable genes and regress out sequencing and experimental batch, mitochondrial mapping percentage, number of UMIs, and number of genes. After filtering and normalization, there were 26,797 cells and 14,953 genes. Following PCA, 5 principle components were selected for clustering tSNE dimensionality reduction.

### Narcolepsy patient TCR sequences

All narcolepsy patient TCR sequences were acquired from the D. Latorre, et al. 2018 study [15].

### Calculation of Levenshtein similarities

L-sim scores were calculated using the levenshteinSim function in the RecordLinkage package for R [24]. L-sim calculation incorporates the Levenshtein distance algorithm, which quantifies the number of edits, deletions, or insertions required to make two strings identical. L-sim includes a string length normalization and transformation of the final value to be between 0 and 1, with 1 representing identical TCR sequences:

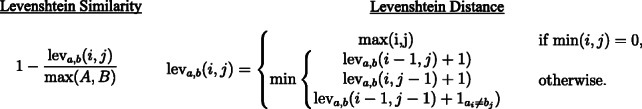



### TCR network visualization

TCR networks that show connections between similar TCRs organized by patient IDs and diagnosis groups were generated using the qgraph function in the qgraph package for R [25]. Analysis from Fig. 3b-d include only clonal TCRs with unambiguous alpha and beta chains. Supplementary Figure 4 includes only clonal TCRs with unambiguous beta chains.

### TCR motif analysis

A custom script was used to identify motifs in our TCR beta chains. Only clonal TCRs with unambiguous beta chains were included for analysis. Identified motifs were searched in the McPAS-TCR database, a manually curated database of TCR sequences found to be associated with pathological conditions in mice and humans [21].

## Supplementary Information

**Additional file 1: Figure S1**. Identification of blood contamination in CSF. a) Representative CSF pellet with visible blood contamination. Samples contaminated with blood should be discarded for molecular analysis of CSF cells. **Figure S2**. Visualization of sample metadata. a) Quality control metrics of each sample after removing low quality samples CSF11 and CSF22 b) tSNE colored by sample. c) tSNE colored by diagnosis. d) tSNE colored by processing batch. e) tSNE colored by sequencing batch. f) Quantification of average cell type distribution per diagnosis based on Seurat clustering. **Figure S3**. Levenshtein Similarity distributions and Identical TCRβ Network. a) Distributions of L-sim scores of clonal full length TCRαβ and TCRβ sequences. b) Network displaying connections between samples with identical TCRβ sequences (L-sim = 1.0). Network includes only clonal TCRs with unambiguous CDR3b sequences. **Figure S4**. Networks of narcolepsy CSF T cells showing shared TCRβ sequences. a) TCRβ network displaying connections between narcolepsy patient samples with identical CD4^+^ T cell TCRβ sequences. Narcolepsy patient (NP) nodes contain clonal CSF CD4^+^ T cell TCRs, healthy control (HC) nodes contain clonal CSF T cells from Supplementary Figure 3b, while HCRT and TRIB2 nodes contain TCRs from peripheral blood derived CD4^+^ T cells that were experimentally determined to be specific for HCRT and TRIB2, respectively. Table below shows metadata for highlighted identical TCRs. Note that there are additional TCRs shared among narcolepsy patients, yet it is unknown whether these TCRs recognize HCRT or another antigen. b) TCRβ network displaying connections between narcolepsy patient CSF samples with identical CD8^+^ T cell TCRβ sequences. All nodes contain clonal CSF CD8^+^ T cell TCRs. HC samples from Supplementary Figure 3b were used. Table below shows metadata for highlighted identical TCRs.

## Data Availability

The datasets analyzed in this study are available in the Gene Expression Omnibus repository under accession number GSE134578. The flow cytometry data generated in this study are available from the corresponding author upon request. The R scripts for TCR similarity analysis are available on github: https://github.com/hamiltonoh/TCR_similarity_analysis.git

## Abbreviations

TCR: T cell receptor
cerebrospinal fluid: CSF
single cell RNA-TCR sequencing: scRNA-TCRseq
AD: Alzheimer’s disease
MCI: Mild cognitive impairment
PD: Parkinson’s disease
HC: Healthy control
NK: Natural killer
DCs: Dendritic cells
L-sim: Levenshtein similarity
EBV: Epstein-Barr virus
