# Supplementary figures and images for: Methods to investigate intrathecal adaptive immunity in neurodegeneration
Source: Mol Neurodegener. 2021 Jan 22;16:3. doi: 10.1186/s13024-021-00423-w (PMC7824942; doi:10.1186/s13024-021-00423-w)

**a** Blood Contamination

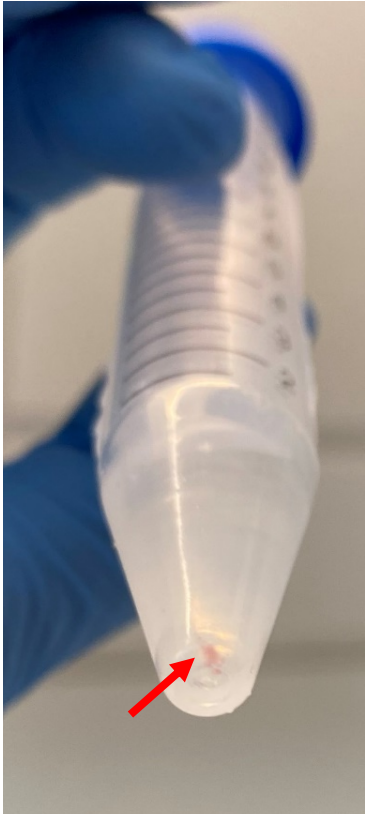

# Supplementary Figure 2

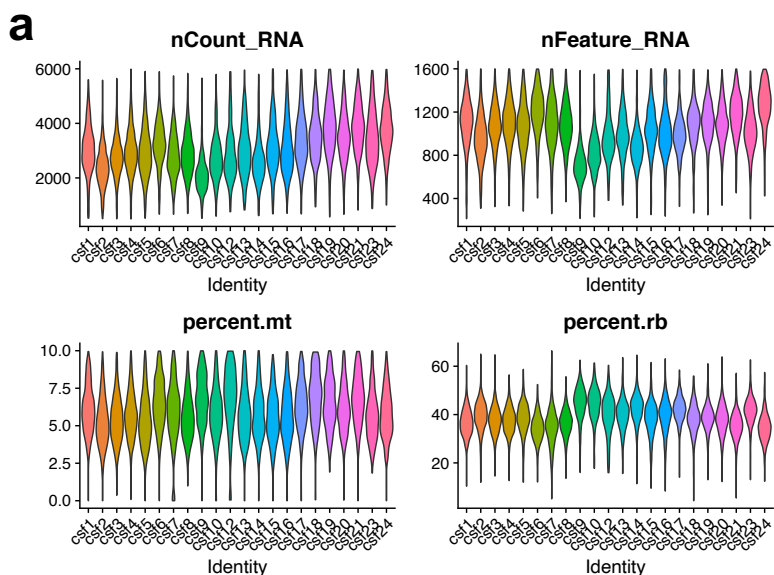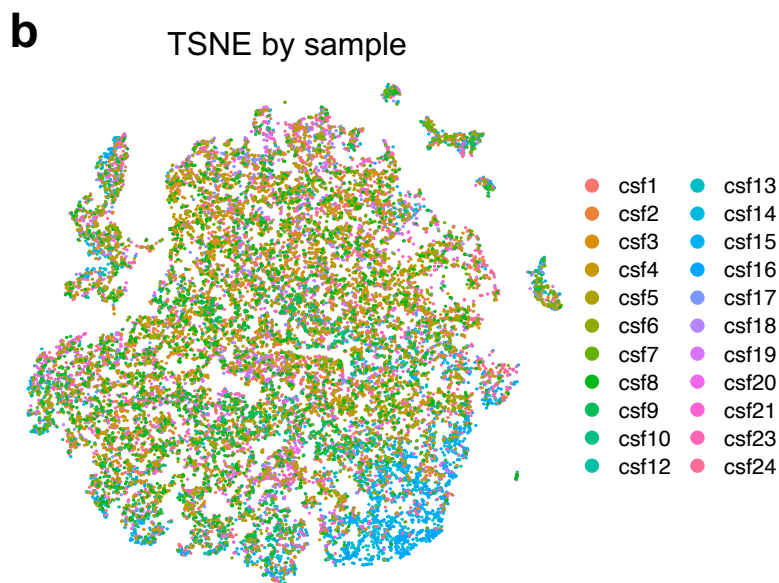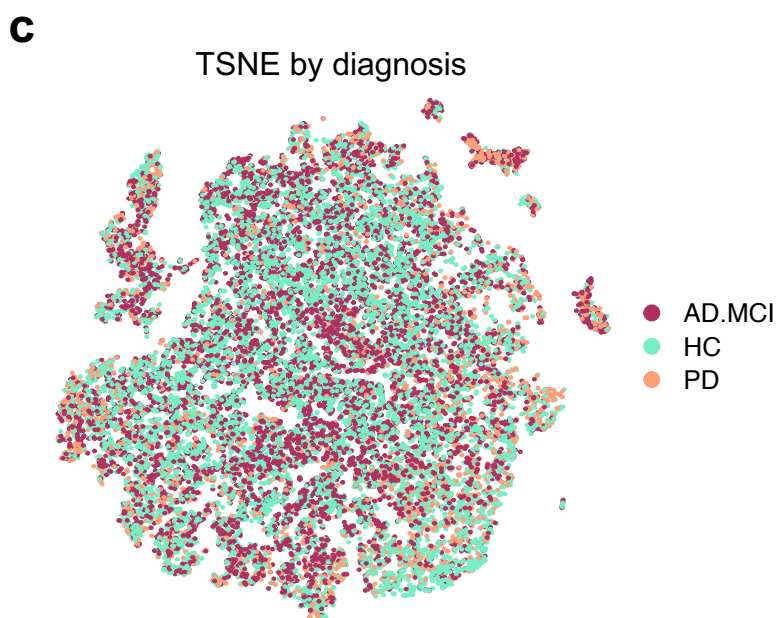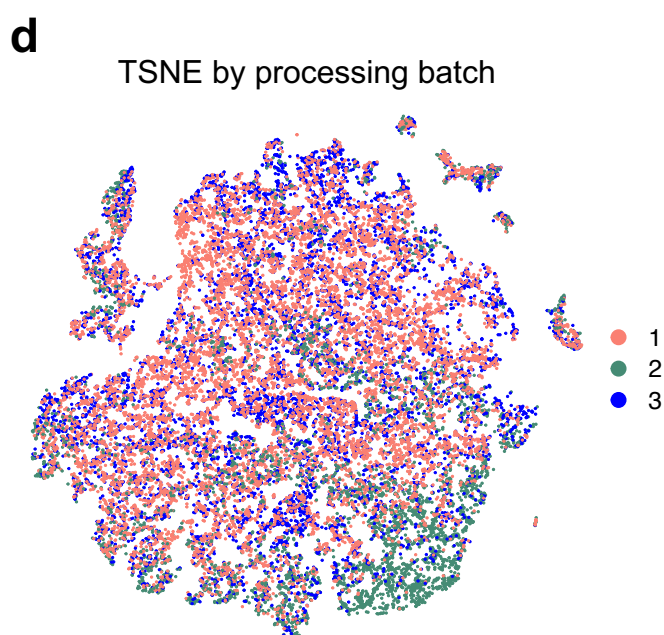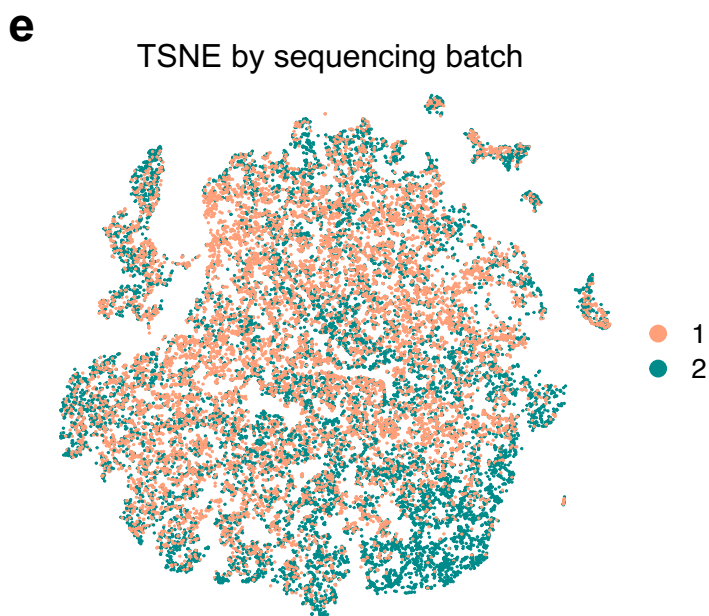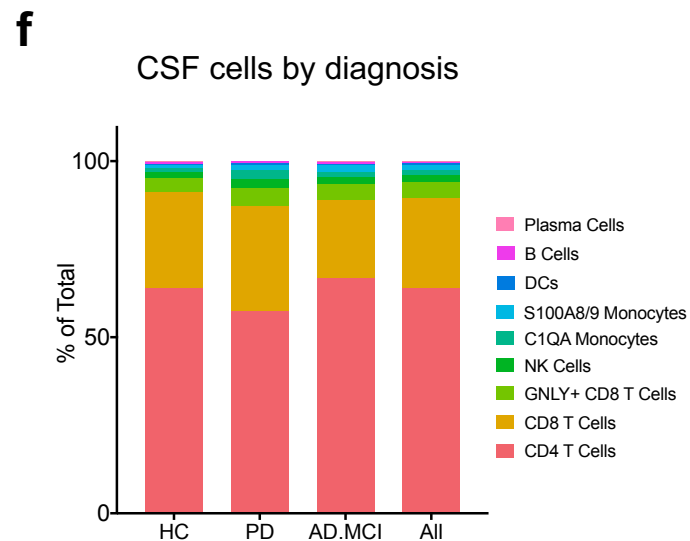

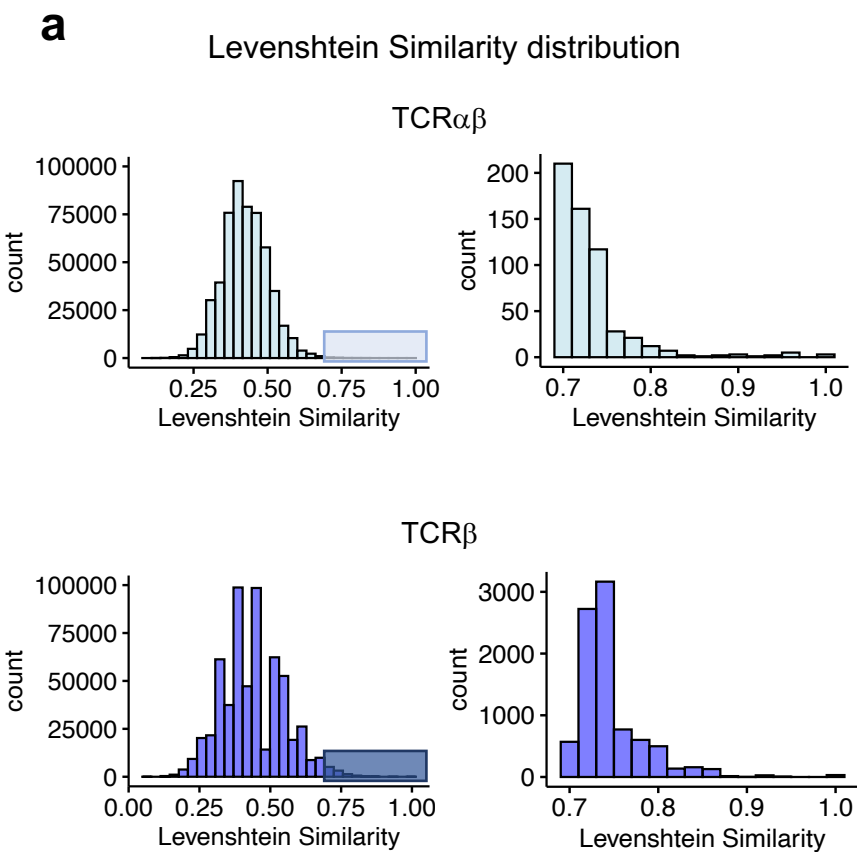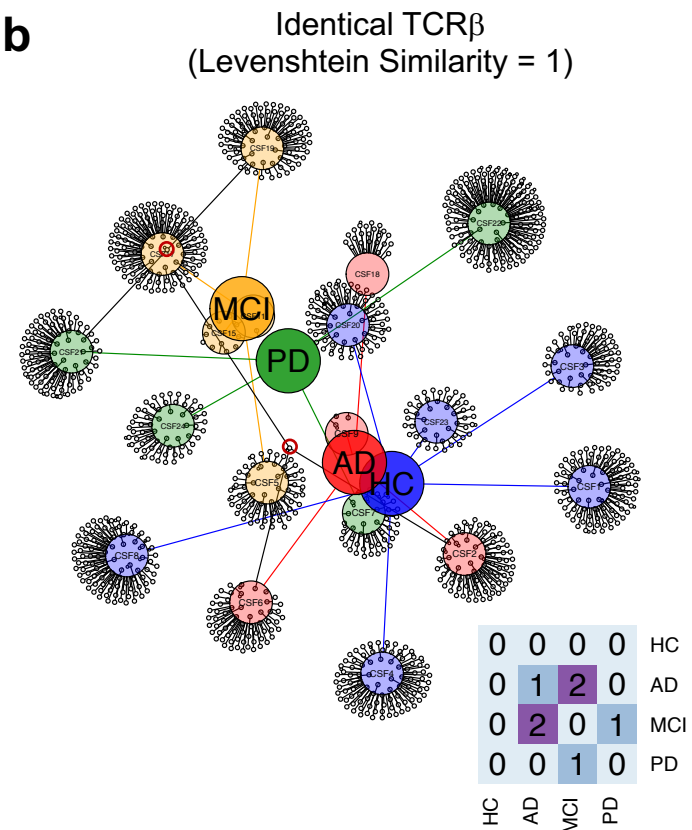

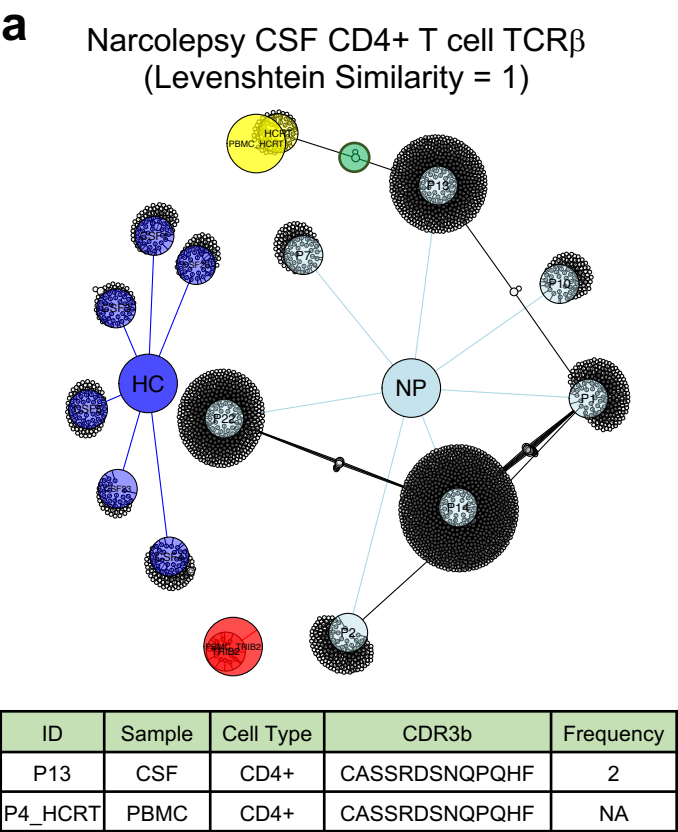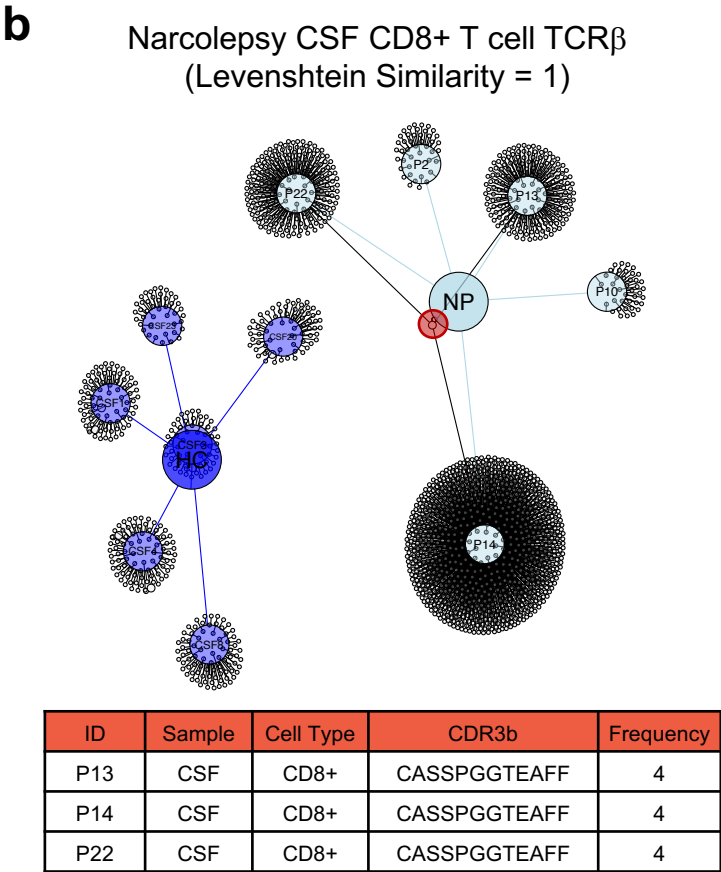

Supplement: Supplementary file 1 — Additional file 1: Figure S1. Identification of blood contamination in CSF. a) Representative CSF pellet with visible blood contamination. Samples contaminated with blood should be discarded for molecular analysis of CSF cells. Figure S2. Visualization of sample metadata. a) Quality control metrics of each sample after removing low quality samples CSF11 and CSF22 b) tSNE colored by sample. c) tSNE colored by diagnosis. d) tSNE colored by processing batch. e) tSNE colored by sequencing batch. f) Quantification of average cell type distribution per diagnosis based on Seurat clustering. Figure S3. Levenshtein Similarity distributions and Identical TCRβ Network. a) Distributions of L-sim scores of clonal full length TCRαβ and TCRβ sequences. b) Network displaying connections between samples with identical TCRβ sequences (L-sim = 1.0). Network includes only clonal TCRs with unambiguous CDR3b sequences. Figure S4. Networks of narcolepsy CSF T cells showing shared TCRβ sequences. a) TCRβ network displaying connections between narcolepsy patient samples with identical CD4+ T cell TCRβ sequences. Narcolepsy patient (NP) nodes contain clonal CSF CD4+ T cell TCRs, healthy control (HC) nodes contain clonal CSF T cells from Supplementary Figure 3b, while HCRT and TRIB2 nodes contain TCRs from peripheral blood derived CD4+ T cells that were experimentally determined to be specific for HCRT and TRIB2, respectively. Table below shows metadata for highlighted identical TCRs. Note that there are additional TCRs shared among narcolepsy patients, yet it is unknown whether these TCRs recognize HCRT or another antigen. b) TCRβ network displaying connections between narcolepsy patient CSF samples with identical CD8+ T cell TCRβ sequences. All nodes contain clonal CSF CD8+ T cell TCRs. HC samples from Supplementary Figure 3b were used. Table below shows metadata for highlighted identical TCRs. [file 13024_2021_423_MOESM1_ESM.pdf]
